# Complete remission of advanced low-grade endometrial stromal sarcoma after aromatase inhibitor therapy: a case report

**DOI:** 10.1186/s13256-021-02838-x

**Published:** 2021-05-05

**Authors:** Omar F. Altal, Ahmed H. Al Sharie, Omar M. Halalsheh, Nour Tashtush, Sarat Shaban, Mahmoud Alfaqih, Abdelwahab Aleshawi

**Affiliations:** 1 Department of Obstetrics & Gynecology, Faculty of Medicine, Jordan University of Science and Technology, King Abdullah University Hospital, P. O. Box: 3030, Irbid, 22110 Jordan; 2 Faculty of Medicine, Jordan University of Science & Technology, Irbid, 22110 Jordan; 3 Department of General Surgery and Urology, Faculty of Medicine, Jordan University of Science & Technology, Irbid, 22110 Jordan; 4 Intern, King Abdullah University Hospital, Jordan University of Science & Technology, Irbid, 22110 Jordan; 5 Department of Physiology, Faculty of Medicine, Jordan University of Science & Technology, Irbid, Jordan

**Keywords:** Endometrial stromal sarcoma, Case report, Hormonal therapy, Letrozole, Complete remission

## Abstract

**Background:**

Low-grade endometrial stromal sarcoma is a rare neoplastic growth in the uterine cavity, representing less than 1% of uterine tumors. Such tumors usually affect premenopausal and perimenopausal women, with a mean age of 46 years. Treatment generally starts with surgical resection of the tumor, followed by chemotherapy, radiotherapy, or hormonal therapy.

**Case presentation:**

In the current report, we again present a case of low-grade endometrial stromal sarcoma in a 51-year-old Mediterranean woman presenting with abdominopelvic pain. Computed tomography scan revealed a primary uterine tumor measuring 17 × 9 × 9 cm metastasizing to the lungs, bladder, and ureteral orifice, along with lymphovascular involvement. The patient underwent total abdominal hysterectomy, omentectomy, and lymph node dissection. Estrogen deprivation was accomplished by bilateral salpingo-oophorectomy. Lifelong hormonal therapy consisting of letrozole 2.5 mg per day was prescribed, which demonstrated remarkable efficacy, resulting in a partial remission of lung metastasis within 8 months after surgery. Full remission was observed after 18 months of hormonal therapy, with no recurrence. Another scan was performed after 2.5 years, revealing complete remission with no recurrence.

**Conclusion:**

We again report a case of complete remission of low-grade endometrial stromal sarcoma after surgical removal of the tumor along with first-line hormonal therapy without the use of chemotherapy or radiotherapy, emphasizing the role of hormonal therapy in the treatment of such tumors.

## Background

Endometrial stromal sarcoma (ESS) is a rare neoplastic growth originating from the mesenchymal elements of the endometrium [1]. It usually affects women between the ages of 40 and 50 [2]. ESS are characterized by their aggressiveness, poor prognosis, and high recurrence rate [3]. Low-grade ESS is associated with better prognosis than high-grade ESS, with higher survival rates [4]. Histological features of ESS include a heavily packed cellular population of oval, round, or spindle-like structures with small thick-walled blood vessels [5]. Several immunohistochemical markers have been identified for the diagnosis of ESS including vimentin, smooth muscle actin (SMA), desmin, S-100, ionized calcium-binding adaptor molecule 1 (IBA1), and CD10 [6]. The detection of YWHAE-FAM22 translocation by molecular studies augmented with immunohistochemistry can differentiate between high- and low-grade ESS, and has replaced the use of mitotic indices as a differentiation parameter [7]. The prognostic factors of ESS are still controversial, and mainly depend on the stage at the time of presentation as assessed by the International Federation of Gynecology and Obstetrics (FIGO) staging of ESS [8]. Treatment typically starts with surgical removal of the tumor, and adjuvant methods including chemotherapy, radiotherapy, and hormonal therapy can be utilized [8]. In the current report, we describe the complete remission of metastasized low-grade ESS after surgical removal of the neoplasm followed by hormonal therapy. To the best of our knowledge, few cases have been reported of complete remission of advanced metastasized low-grade ESS after surgical and hormonal therapy alone, without the need for chemotherapy or radiotherapy. This case report was written in accordance with the CARE guidelines for case reports [9].

## Case presentation

A 51-year-old married Mediterranean woman with a nonsignificant past medical history presented to our center through the outpatient clinic, complaining of menorrhagia and abdominal pain for the past 2 weeks. The pain was radiating to the back and was associated with loss of appetite and stress incontinence. The patient reported no episodes of dysmenorrhea. She had no remarkable surgical history prior to her current symptoms and no family history of malignancies. The physical examination revealed no significant findings. Ultrasonography revealed multiple uterine masses and polyps along with an enlarged uterus and thin endometrial lining.

**Fig. 1:**
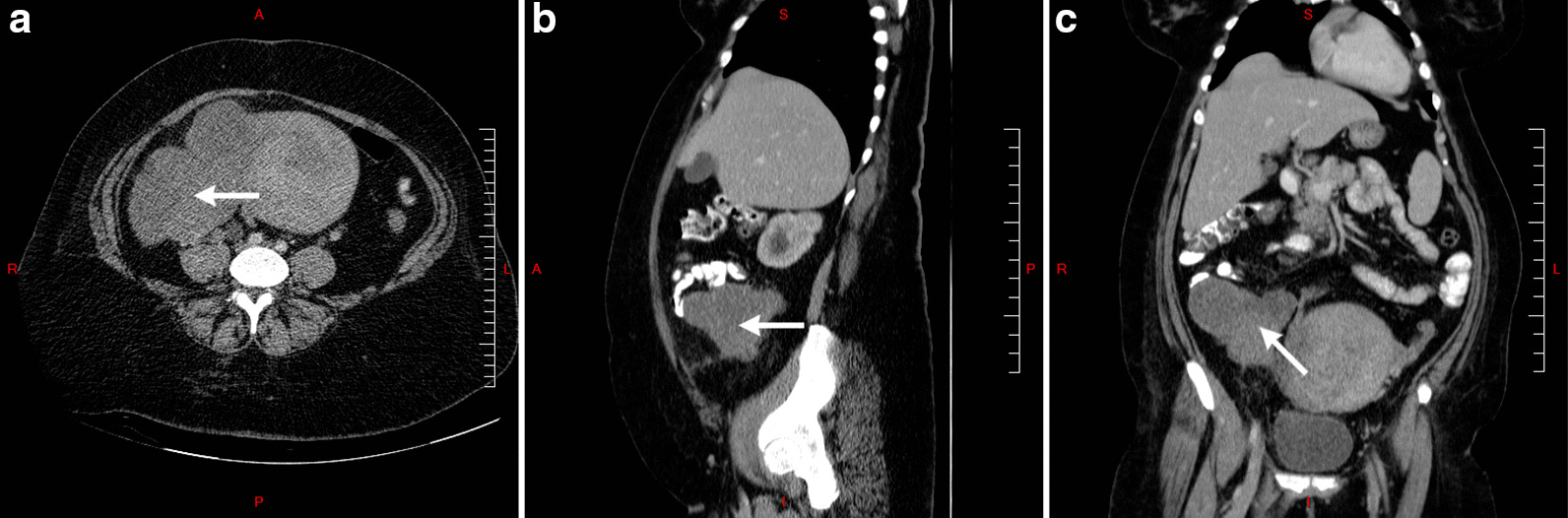
Transverse (**a**), sagittal (**b**), and coronal (**c**) computed tomography scans showing a primary uterine mass as pointed by the white arrow

Basic laboratory investigations were performed and all were within the normal range except for complete blood count, which indicated microcytic hypochromic anemia. She was suspected of having uterine fibroids. Accordingly, hysteroscopy along with dilation and curettage (D&C) was performed. Operative findings included a 22-week-sized uterus that was deviated to the left side. In addition, cystocele and rectocele were noted, both assessed as grade II. Hysteroscopy findings included multiple uterine fibroids and polyps. Endometrial biopsy revealed infarcted endometrial polyps. Plasma tumor markers were evaluated to rule out any possible malignancies. Results indicated high levels of lactate dehydrogenase (LDH: 441 U/L), alpha-fetoprotein (AFP: 1.9 ng/mL), and CA-125 (106.8 U/mL), along with normal values for beta-human chorionic gonadotropin (β-hCG), cancer antigen (CA) 15-3, CA 19-9, and carcinoembryonic antigen (CEA). Abdominal and pelvic computed tomography (CT) scan showed an irregular hypodense lesion in the uterus containing an area of necrosis measuring 17 × 9 × 9 cm, suggesting a primary uterine tumor (Fig. 1a–c). CT scan also revealed wall thickening in the right posterolateral aspect of the urinary bladder, with filling defect and wall irregularity causing severe hydronephrosis on the right side (Fig. 1b). Enlarged lymph nodes were seen in both the external and internal iliac regions, the largest measuring 1.3 cm.

**Fig. 2:**
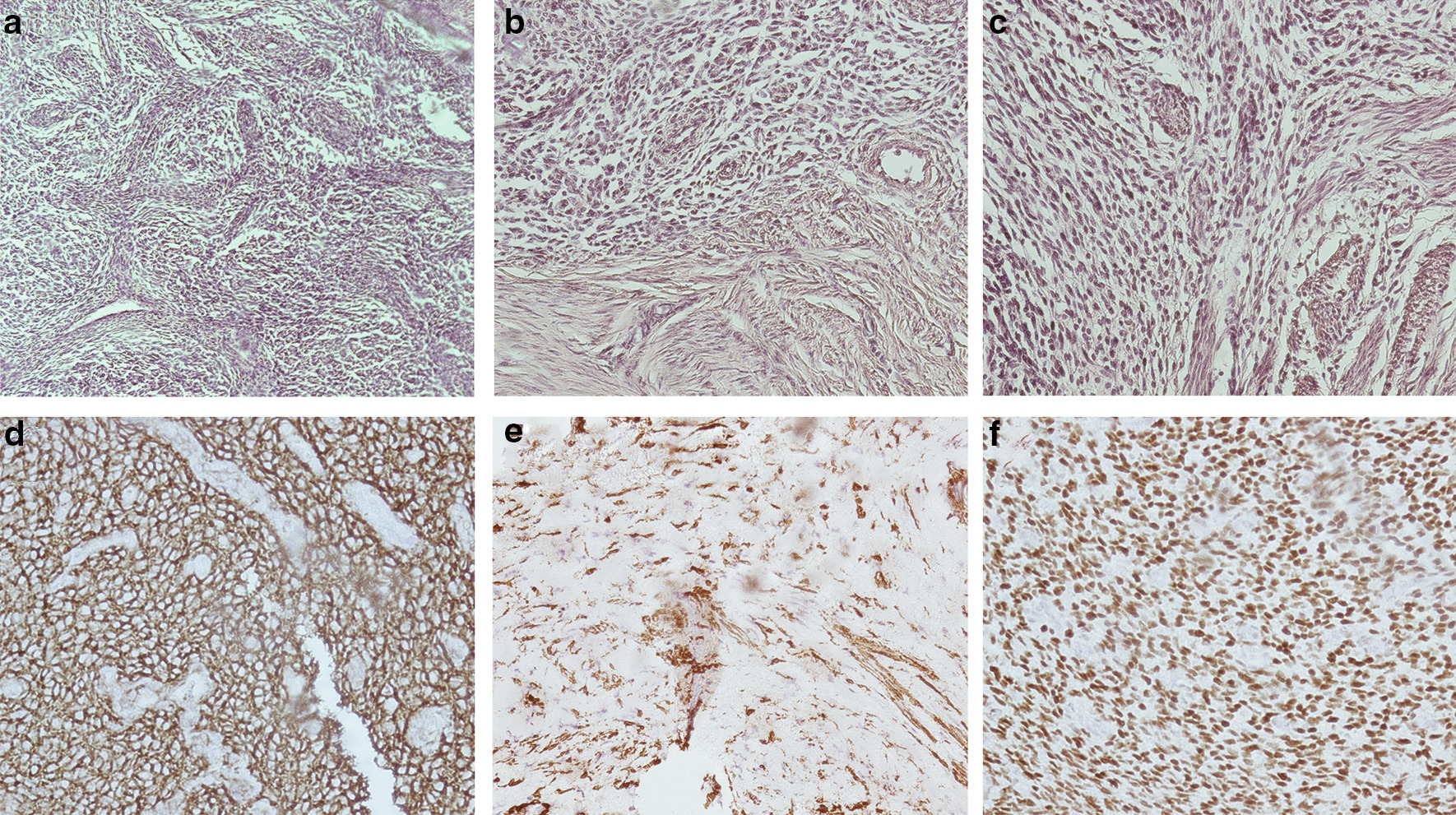
Histopathological investigations of six true-cut biopsies for the same mass seen on computed tomography scan. Hematoxylin and eosin (H&E) stains for different samples (**a**–**c**) illustrating hypercellular spindle cell lesions, with the nuclei of some cells resembling those of smooth muscle origin, with mild cellular pleomorphism and nonsignificant count of mitotic figures. The cellular population was positive for CD10 (**d**), smooth muscle actin (**e**), and estrogen receptor (**f**)

Ultrasonography-guided biopsy under aseptic conditions with the application of local anesthetic was used to take six true-cut biopsies for the same mass seen on the CT scan. Histopathological examination showed a hypercellular spindle cell lesion, with some of the cells having nuclei resembling those of smooth muscle origin (Fig. 2a–c). Mild cellular pleomorphism along with nonsignificant count of mitotic figures was observed. Thick-walled spindly blood vessels were seen in the background. Immunohistochemical studies revealed that the spindle cell population was positive for CD10, SMA, and estrogen receptor (ER) but negative for CD34, CD117, epithelial membrane antigen (EMA), and desmin, as shown in Fig. 2d–f. A diagnosis of low-grade ESS was established.

**Fig. 3:**
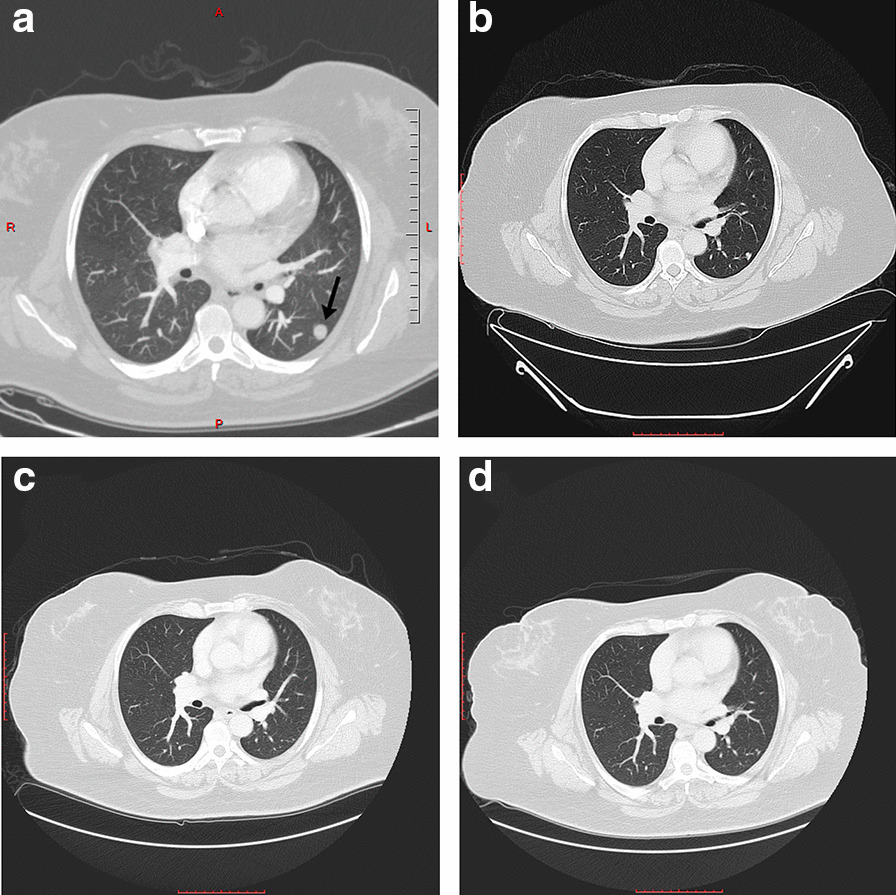
Transverse computed tomography image showing lung metastasis as pointed by the black arrow (**a**). Partial remission of the metastasized tumor was achieved after 8 months of letrozole 2.5 mg per day (**b**). Complete remission was observed after 18 months (**c**) and 2.5 years (**d**) with the same therapeutic regimen

Subsequently, right double-J (DJ) stent insertion was performed. The operative findings included a mass around the right ureteral orifice with multiple varicosities and neovascularization around and in the bladder, respectively. Microscopic examination of bladder biopsy showed fragments of urothelial mucosa infiltrated by nodules of small monotonous round cell tumor. The tumor cells were immunoreactive against CD10 and ER but negative for keratin 20 (CK20), CD34, cyclin D1, and SMA. The results were consistent with the diagnosis of ESS. Chest CT scan showed a left lower lobe nodule peripherally mostly representing a malignant deposit (Fig. 3a). Pulmonary embolism (PE) in the secondary and tertiary branches of the right pulmonary artery was observed incidentally, with elevated D-dimer (4.1 μg/mL) and fibrinogen (598 mg/dL). In addition, CT angiogram confirmed the diagnosis of acute PE. The patient started therapeutic doses of enoxaparin (100 mg, every 12 hours), after which she underwent staging laparotomy. Operative findings included uterus (22 weeks in size) with tumor invading the right fallopian tube and adherent to the right pelvic side wall and the right side of the bladder wall. Total abdominal hysterectomy (TAH) and bilateral salpingo-oophorectomy (BSO), omentectomy, and lymph node dissection were performed. Biopsies were taken from the uterus, both ovaries, both fallopian tubes, omentum, and right external iliac lymph node, which all confirmed the diagnosis of low-grade ESS with lymphovascular invasion.

**Fig. 4:**
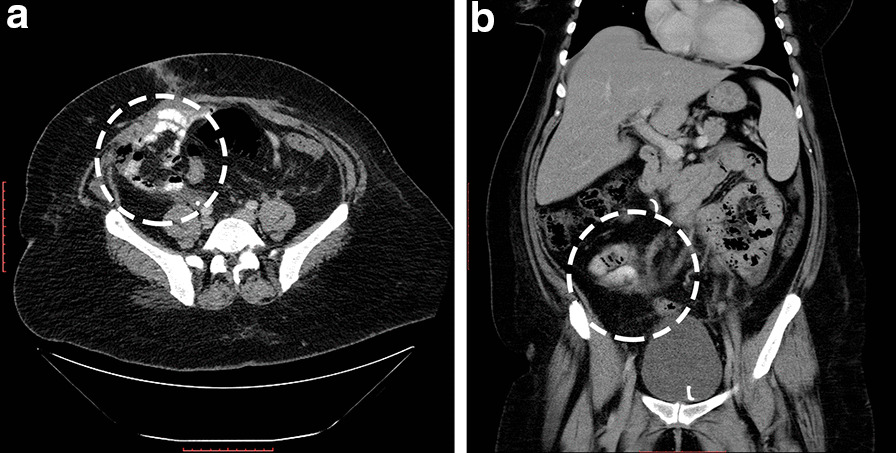
Transverse (**a**) and coronal (**b**) computed tomography images showing full neoplastic remission after 18 months of letrozole 2.5 mg per day without any evidence of a primary uterine tumor marked by the dashed circle

Hormonal therapy with letrozole 2.5 mg daily was initiated. The patient was then discharged with a 10-day course of clindamycin and lifelong enoxaparin treatment. She was followed up in the outpatient clinic with right DJ stent exchange every 3 months. Partial remission of lung metastasis was achieved after 8 months of hormonal therapy, which was confirmed by a clean CT image (Fig. 3b). After 18 months, another chest CT was conducted and revealed complete resolution of the metastasis (Fig. 3c). Another chest and abdominopelvic CT scan were performed after 2.5 years form the surgery, which also confirmed the full remission of ESS as shown in Fig. 3d for the lung metastasis and Fig. 4a, b for the primary lesion.

## Discussion

ESS is an extremely rare uterine malignancy, constituting 0.2% of all genital tract neoplasms, with an incidence of 2 per million women worldwide [10]. Such uterine tumors are aggressive in nature and associated with poor prognosis and high rates of recurrence, and can affect women of both reproductive and postmenopausal age [3]. According to the World Health Organization (WHO), ESS is categorized into low- and high-grade ESS along with nodular and undifferentiated forms of the neoplasm; the classification is based on the morphology and the prognosis of the tumor as well as cellular cytogenetic properties [7].

Clinical presentation of ESS varies according to the affected area, but patients commonly complain of abdominal pain, vaginal bleeding, vomiting, diarrhea, constipation, hematuria, and increased urinary frequency and urgency with incontinence [11]. The pathogenesis of ESS includes gene rearrangement, loss of tumor suppressor genes, microsatellite instability, and loss of heterozygosity [12]. Some reports suggest that prolonged exposure to endogenous or exogenous estrogens along with tamoxifen treatment can play an important role in the development of the tumor [13, 14].

Treatment for ESS starts with surgical removal of the neoplasm and the associated invaded tissues [8]. Since the histopathological profile suggests a nonsignificant count of mitotic figures, the neoplasm will respond poorly to chemotherapeutic agents [15]. However, responsiveness to chemotherapy was described previously in a reported case suggesting total remission of ESS following a combination of chemotherapeutic agents [16]. On the other hand, some studies suggest a beneficial outcome for radiotherapy against high-grade ESS, while others report a nonsignificant effect against low- or high-grade forms of ESS [15, 17]. ESS can express estrogen receptor (ER) and progesterone receptors (PR), which can be effectively used as a target to suppress the growth of the neoplasm [18]. The suggested protocol is treatment with aromatase inhibitors with or without gonadotropin-releasing hormone (GnRH) if the cellular population is ER-positive; if the tumor is positive for both ER and PR, progestins can be used [19]. GnRH is used without any combination if the tumor is positive only for PR [19]. Estrogen deprivation after BSO can be very useful, especially for metastasized ESS [20]. Hormonal therapy has proved to be an effective tool against ESS, with improved long-term survival [21, 22]. Several chemotherapeutic regimens have been introduced for the treatment of ESS including carboplatin or gemcitabine plus docetaxel [23, 24]. In addition, pazopanib, a selective multi-targeted receptor tyrosine kinase inhibitor, has been shown to be an efficient agent for ESS [25–27].

Our patient complained of abdominal pain and menorrhagia, which are typical presentations for ESS, and since she was in her early 50s, she was prone to such neoplastic growth [2]. What is unique about our case is that the patient experienced a full neoplastic remission after a combination of surgical removal of the tumor and hormonal therapy including estrogen deprivation by BSO and a course of letrozole as previously described. Despite ESS aggressiveness and the extensive metastatic spread across the bladder, ureteral orifice, and lung with lymphovascular involvement, full remission was observed 18 weeks after surgery, which was confirmed by a clear CT scan. Several cases reports have demonstrated the importance of hormonal therapy in the management of ESS. Alkasi *et al*. reported long-term survival of a 28-year-old patient with metastatic ESS treated with goserelin and anastrozole after surgical intervention [28]. A similar report by Spano *et al*. described total response in two women with ESS and lung metastasis after surgical removal of the tumor followed by treatment with aminoglutethimide, an aromatase inhibitor [21]. Leunen *et al*. described outstanding response to letrozole in a 76-year-old patient with a huge pelvic mass accompanied by post-renal kidney failure [29]. Hormonal therapy has also proved effective in the case of neoplastic recurrence, where Shoji *et al*. reported that anastrozole improved prognosis in a case of recurrent low-grade ESS in a 34-year-old woman [30]. Hormonal therapy has also been used to shrink the tumor before surgical resection, as reported in the case of a 47-year-old woman with low-grade ESS [31]. Despite complete remission of ESS, such tumors carry a high risk of recurrence even after two decades of treatment, as reported by Gangireddy *et al*., which highlights the importance of regular follow-ups [32].

We report yet another case of complete neoplastic remission of low-grade ESS with extensive metastasis to the lungs, bladder, and ureter along with lymphovascular involvement after surgical and hormonal therapy, in hopes of expanding the knowledge about such rare neoplasms.

## Conclusion

In the current case, we reported a 51-year-old woman diagnosed with low-grade ESS, where the neoplasm had started as a primary uterine tumor measuring 17 × 9 × 9 cm and had metastasized to the bladder, ureteral orifice, and external and internal iliac lymph nodes. Malignant depositions were also observed in the lung. The patient achieve total remission of the neoplasm, which suggests an excellent synergistic effect between surgical intervention and hormonal therapy that includes letrozole 2.5 mg per day. Further studies and trials are needed to establish a protocol for treatment of such tumors.

## Data Availability

Not applicable.

## Abbreviations

ESS: Endometrial stromal sarcoma
SMA: Smooth muscle actin
IBA1: Ionized calcium-binding adaptor molecule 1
FIGO: International Federation of Gynecology and Obstetrics
D&C: Dilation and curettage
LDH: Lactate dehydrogenase
AFP: Alpha-fetoprotein
β-hCG: Beta-human chorionic gonadotropin
CEA: Carcinoembryonic antigen
CT: Computed tomography
ER: Estrogen receptor
PR: Progesterone receptor
DJ: Double-J
PE: Pulmonary embolism
TAH: Total abdominal hysterectomy
BSO: Bilateral salpingo-oophorectomy

## References

[1] Dahhan T, Fons G, Buist MR, ten Kate FJW, van der Velden J. The efficacy of hormonal treatment for residual or recurrent low-grade endometrial stromal sarcoma. A retrospective study. Eur J Obstet Gynecol Reprod Biol. 2009;144(1):80–4. 10.1016/j.ejogrb.2009.02.005 19269732

[2] Puliyath G, Nair VR, Singh S. Endometrial stromal sarcoma. Indian J Med Paediatr Oncol. 2010;31(1):21–3. 10.4103/0971-5851.68848 PMC2941598 20931016

[3] Rauh-Hain JA, del Carmen MG. Endometrial stromal sarcoma: a systematic. Obstetr Gynecol. 2013;122(3):676–83. 10.1097/AOG.0b013e3182a189ac 23921879

[4] Seagle B-LL, Shilpi A, Buchanan S, Goodman C, Shahabi S. Low-grade and high-grade endometrial stromal sarcoma: a national cancer database study. Gynecol Oncol. 2017;146(2):254–62. 10.1016/j.ygyno.2017.05.036 28596015

[5] Young RH, Prat J, Scully RE. Endometrioid stromal sarcomas of the ovary. A clinicopathologic analysis of 23 cases. Cancer. 1984;53(5):1143–55. 10.1002/1097-0142(19840301)53:5<1143::aid-cncr2820530521>3.0.co;2-f 6198065

[6] Kumabe S, Sato J, Tomonari Y, Takahashi M, Inoue K, Yoshida M, Doi T, Wako Y, Tsuchitani M. Morphological and immunohistochemical diversity of endometrial stromal sarcoma in rats. J Toxicol Pathol. 2018;31(2):95–103. 10.1293/tox.2017-0056 PMC5938210 29749998

[7] Conklin CMJ, Longacre TA. Endometrial stromal tumors: the new WHO classification. Adv Anat Pathol. 2014;21(6):383–93. 10.1097/PAP.0000000000000046 25299308

[8] Puliyath G, Nair MK. Endometrial stromal sarcoma: a review of the literature. Indian J Med Paediatr Oncol. 2012;33(1):1–6. 10.4103/0971-5851.96960 PMC3385272 22754201

[9] Riley DS, Barber MS, Kienle GS, Aronson JK, von Schoen-Angerer T, Tugwell P, Kiene H, Helfand M, Altman DG, Sox H,* et al*. CARE guidelines for case reports: explanation and elaboration document. J Clin Epidemiol. 2017;89:218–35. 10.1016/j.jclinepi.2017.04.026 28529185

[10] Chu MC, Mor G, Lim C, Zheng W, Parkash V, Schwartz PE. Low-grade endometrial stromal sarcoma: hormonal aspects. Gynecol Oncol. 2003;90(1):170–6. 10.1016/s0090-8258(03)00258-0 12821359

[11] Masand RP. Unusual presentations of gynecologic tumors: primary, extrauterine, low-grade endometrioid stromal sarcoma. Arch Pathol Lab Med. 2018;142(4):536–41. 10.5858/arpa.2017-0241-RA 29293021

[12] Micci F, Heim S. Pathogenetic mechanisms in endometrial stromal sarcoma. Cytogenet Genome Res. 2007;118(2–4):190–5. 10.1159/000108300 18000370

[13] Altaras MM, Jaffe R, Cohen I, Gruber A, Yanai-Inbar I, Bernheim J. Role of prolonged excessive estrogen stimulation in the pathogenesis of endometrial sarcomas: two cases and a review of the literature. Gynecol Oncol. 1990;38(2):273–7. 10.1016/0090-8258(90)90054-o 2167285

[14] Hu R, Hilakivi-Clarke L, Clarke R. Molecular mechanisms of tamoxifen-associated endometrial cancer (Review). Oncol Lett. 2015;9(4):1495–501. 10.3892/ol.2015.2962 PMC4356269 25788989

[15] Stewart LE, Beck TL, Giannakopoulos NV, Rendi MH, Isacson C, Goff BA. Impact of oophorectomy and hormone suppression in low grade endometrial stromal sarcoma: a multicenter review. Gynecol Oncol. 2018;149(2):297–300. 10.1016/j.ygyno.2018.03.008 29534832

[16] Lehrner LM, Miles PA, Enck RE. Complete remission of widely metastatic endometrial stromal sarcoma following combination chemotherapy. Cancer. 1979;43(4):1189–94. 10.1002/1097-0142(197904)43:4<1189::aid-cncr2820430405>3.0.co;2-e 445321

[17] Barney B, Tward JD, Skidmore T, Gaffney DK. Does radiotherapy or lymphadenectomy improve survival in endometrial stromal sarcoma? Int J Gynecol Cancer. 2009;19(7):1232–8. 10.1111/IGC.0b013e3181b33c9a 19823060

[18] Navarro D, Cabrera JJ, León L, Chirino R, Fernández L, López A, Rivero JF, Fernández P, Falcón O, Jiménez P,* et al*. Endometrial stromal sarcoma expression of estrogen receptors, progesterone receptors and estrogen-induced srp27 (24K) suggests hormone responsiveness. J Steroid Biochem Mol Biol. 1992;41(3–8):589–96. 10.1016/0960-0760(92)90389-z 1562530

[19] Reich O, Regauer S. Hormonal therapy of endometrial stromal sarcoma. Curr Opin Oncol. 2007;19(4):347–52. 10.1097/CCO.0b013e3281a7ef3a 17545798

[20] Thanopoulou E, Aleksic A, Thway K, Khabra K, Judson I. Hormonal treatments in metastatic endometrial stromal sarcomas: the 10-year experience of the sarcoma unit of Royal Marsden Hospital. Clin Sarcoma Res. 2015;5(1):8. 10.1186/s13569-015-0024-0 PMC4373094 25810898

[21] Spano JP, Soria JC, Kambouchner M, Piperno-Neuman S, Morin F, Morere JF, Martin A, Breau JL. Long-term survival of patients given hormonal therapy for metastatic endometrial stromal sarcoma. Med Oncol. 2003;20(1):87. 10.1385/MO:20:1:87 12665689

[22] Deshmukh U, Black J, Perez-Irizarry J, Passarelli R, Levy K, Rostkowski A, Hui P, Rutherford TJ, Santin AD, Azodi M,* et al*. Adjuvant hormonal therapy for low-grade endometrial stromal sarcoma. Reprod Sci. 2019;26(5):600–8. 10.1177/1933719118778801 29843577

[23] Szlosarek PW, Lofts FJ, Pettengell R, Carter P, Young M, Harmer C. Effective treatment of a patient with a high-grade endometrial stromal sarcoma with an accelerated regimen of carboplatin and paclitaxel. Anticancer Drugs. 2000;11(4):275–8. 10.1097/00001813-200004000-00008 10898543

[24] Takano T, Niikura H, Ito K, Nagase S, Utsunomiya H, Otsuki T, Toyoshima M, Tokunaga H, Kaiho-Sakuma M, Shiga N,* et al*. Feasibility study of gemcitabine plus docetaxel in advanced or recurrent uterine leiomyosarcoma and undifferentiated endometrial sarcoma in Japan. Int J Clin Oncol. 2014;19(5):897–905. 10.1007/s10147-013-0627-5 24149774

[25] Kim HJ, Kim Y, Lee SJ, Lee J, Park SH. Pazopanib monotherapy in the treatment of pretreated, metastatic uterine sarcoma: a single-center retrospective study. J Gynecol Oncol. 2018;29(1):e3. 10.3802/jgo.2018.29.e3 PMC5709529 29185261

[26] Sunar V, Korkmaz V, Akin S, Can Guven D, Arik Z, Ates O, Yilmaz M, Mutlu Meydanli M, Oksuzoglu B. Efficacy of Pazopanib in patients with metastatic uterine sarcoma: a multi-institutional study. J BUON. 2019;24(6):2327–32. 31983102

[27] Verschoor AJ, Warmerdam FARM, Bosse T, Bovée JVMG, Gelderblom H. A remarkable response to pazopanib, despite recurrent liver toxicity, in a patient with a high grade endometrial stromal sarcoma, a case report. BMC Cancer. 2018;18(1):92. 10.1186/s12885-018-3999-0 PMC5778698 29357824

[28] Alkasi Ö, Meinhold-Heerlein I, Zaki R, Fasching P, Maass N, Jonat W, Beckmann MW. Long-term disease-free survival after hormonal therapy of a patient with recurrent low grade endometrial stromal sarcoma: a case report. Arch Gynecol Obstet. 2009;279(1):57–60. 10.1007/s00404-008-0631-6 18379806

[29] Leunen M, Breugelmans M, De Sutter P, Bourgain C, Amy JJ. Low-grade endometrial stromal sarcoma treated with the aromatase inhibitor letrozole. Gynecol Oncol. 2004;95(3):769–71. 10.1016/j.ygyno.2004.07.063 15582003

[30] Shoji K, Oda K, Nakagawa S, Kawana K, Yasugi T, Ikeda Y, Takazawa Y, Kozuma S, Taketani Y. Aromatase inhibitor anastrozole as a second-line hormonal treatment to a recurrent low-grade endometrial stromal sarcoma: a case report. Med Oncol. 2011;28(3):771–4. 10.1007/s12032-010-9511-6 20354812

[31] Scribner DR, Walker JL. Low-grade endometrial stromal sarcoma preoperative treatment with Depo-Lupron and Megace. Gynecol Oncol. 1998;71(3):458–60. 10.1006/gyno.1998.5174 9887250

[32] Gangireddy M, Chan Gomez J, Kanderi T, Joseph M, Kundoor V. Recurrence of endometrial stromal sarcoma, two decades post-treatment. Cureus. 2020;12(7):e9249. 10.7759/cureus.9249 PMC7430693 32821595

